# Blockade of Persistent Sodium Currents Contributes to the Riluzole-Induced Inhibition of Spontaneous Activity and Oscillations in Injured DRG Neurons

**DOI:** 10.1371/journal.pone.0018681

**Published:** 2011-04-25

**Authors:** Rou-Gang Xie, Da-Wei Zheng, Jun-Ling Xing, Xu-Jie Zhang, Ying Song, Ya-Bin Xie, Fang Kuang, Hui Dong, Si-Wei You, Hui Xu, San-Jue Hu

**Affiliations:** 1 Institute of Neuroscience, Xi Jing Hospital, The Fourth Military Medical University, Xi'an, People's Republic of China; 2 School of Stomatology, Xi Jing Hospital, The Fourth Military Medical University, Xi'an, People's Republic of China; 3 Department of Anaesthiology, Xi Jing Hospital, The Fourth Military Medical University, Xi'an, People's Republic of China; 4 Jiangsu Province Key Laboratory of Anesthesiology, Xuzhou Medical College, Xuzhou, People's Republic of China; University of Cincinnatti, United States of America

## Abstract

In addition to a fast activating and immediately inactivating inward sodium current, many types of excitable cells possess a noninactivating or slowly inactivating component: the persistent sodium current (*I_NaP_*). The *I_NaP_* is found in normal primary sensory neurons where it is mediated by tetrodotoxin-sensitive sodium channels. The dorsal root ganglion (DRG) is the gateway for ectopic impulses that originate in pathological pain signals from the periphery. However, the role of I_NaP_ in DRG neurons remains unclear, particularly in neuropathic pain states. Using *in vivo* recordings from single medium- and large-diameter fibers isolated from the compressed DRG in Sprague-Dawley rats, we show that local application of riluzole, which blocks the *I_NaP_*, also inhibits the spontaneous activity of A-type DRG neurons in a dose-dependent manner. Significantly, riluzole also abolished subthreshold membrane potential oscillations (SMPOs), although DRG neurons still responded to intracellular current injection with a single full-sized spike. In addition, the *I_NaP_* was enhanced in medium- and large-sized neurons of the compressed DRG, while bath-applied riluzole significantly inhibited the *I_NaP_* without affecting the transient sodium current (*I_NaT_*). Taken together, these results demonstrate for the first time that the *I_NaP_* blocker riluzole selectively inhibits I_NaP_ and thereby blocks SMPOs and the ectopic spontaneous activity of injured A-type DRG neurons. This suggests that the *I_NaP_* of DRG neurons is a potential target for treating neuropathic pain at the peripheral level.

## Introduction

Voltage-dependent sodium channels are responsible for the generation and conduction of action potentials in the membranes of excitable cells. In addition to a fast activating and immediately inactivating sodium current, the inward current of many types of excitable cells also has a non-inactivating or slowly inactivating component: the persistent sodium current (*I_NaP_*). The *I_NaP_* is present in neurons throughout the central nervous system, including those of the hippocampus, neocortex and cerebellum [1]; it has also been found in thalamic neurons [1], [2], mesencephalic trigeminal sensory neurons [3], and hypoglossal motoneurons [4]. When present, *I_NaP_* lowers the actviation threshold of most neurons by about 10 mV, and is blocked by a low level of tretrodotoxin (100 nM) [1], [5], [6]. Under physiological conditions, *I_NaP_* is critical to neuronal excitability, the modulation of near-threshold membrane potentials, the amplification of synaptic currents, and the facilitation of repetitive firing [7], [8], [9], [10]. There is evidence from a genetic model of amyotrophic lateral sclerosis and spinal cord injury that an increased persistent sodium current determines the hyperexcitability of central cortical neurons [11], [12]. It also has been reported that *I_NaP_* participates in epileptic firing in the central nervous system [5]. Among such cells as mesencephalic trigeminal sensory neurons and hippocampal neurons, *I_NaP_* is considered to be one of the threshold currents modulating neuronal excitability under both physiological and pathological conditions.

Recently, a number of reports have focused on the effects of riluzole on *I_NaP_* in central neurons [13], [14], [15], [16], [17], and have proposed riluzole as a relatively specific persistent sodium channel blocker [18], [19]. Riluzole has been used clinically in the treatment of several neurological disorders, including amyotrophic lateral sclerosis [20] and epilepsy. However, the mechanisms underlying these clinical applications are far from clear.

The dorsal root ganglion (DRG) is the gateway for ectopic impulses originating in pathological pain signals from the periphery. The *I_NaP_* has been found in normal primary sensory neurons where it is mediated by tetrodotoxin-sensitive sodium channels [21]. However, the role of *I_NaP_* in DRG neurons is uncertain, particularly in neuropathic pain states. Our recent work has shown that in compressed DRG neurons, *I_NaP_* is blocked by gabapentin and low doses of lidocaine, and that these analgesic drugs suppress the submembrane potential oscillations of injured DRG neurons [22], [23].

In the present study we demonstrate that behavioral changes in rats undergoing a chronic compression of the dorsal root ganglion (CCD) [24], [25] are concurrent with a significant enhancement of *I_NaP_*, along with its associated subthreshold membrane potential oscillations (SMPOs) and ectopic spotaneous activity (SA), in the correspondingly injured A-type DRG neurons. Local application of riluzole clearly inhibits *I_NaP_* while suppressing SMPOs and SA, indicating a potential role for the I_NaP_ of injured DRG neurons in neuropathic pain states.

## Materials and Methods

### 1. CCD animal model

Experiments were conducted on adult Sprague–Dawley rats (200–250 g) of both sexes. The animals were purchased from the Animal Center of the Fourth Military Medical University (FMMU) and were housed and handled according to the guidelines of the institutional and national Committees of Animal Use and Protection.

The animal model used in this study was established in our department as described previously [24], [25]. In the present study, a stainless steel L-shaped rod (4 mm in length and 0.66 mm in diameter) was implanted in the intervertebral forman at L5 to chronically compress the DRG.

### 2. Behavioral testing

#### Mechanical paw withdrawal threshold

Paw withdrawal thresholds to mechanical stimulation were assessed using von Frey filaments (Stoelting Co, USA). Each animal was placed on a metal mesh floor in a plastic cage (20×25×15 cm). To test the tactile threshold required to evoke withdrawal of the stimulated paw, von Frey filaments (2–15.0 g) were applied perpendicularly in ascending order to the plantar part of the hind paw [26]. Withdrawal, flicking, or licking of the hind paw were all considered positive responses. Each filament was applied five times, with the overall response assessed as positive if three or more positive responses of the hind paw were obtained. The paw withdrawal threshold was determined by the lowest strength of stimulation. To avoid tissue damage, the cut off threshold was assigned at 15.0 g [27].

### 3. Electrophysiological recordings

#### 3.1 Extracellular recording of DRG single fiber activities

Unit activities of single DRG A-fibers were recorded 3–8 days after the CCD surgery. Under sodium pentobabital anesthesia (40 mg/kg, i.p.), laminectomies were performed at the L1–L2 and L4–L5 levels separately, and two small pools were formed above the exposure regions. In the L4–L5 pool the stainless steel rod was removed. The spinal nerve was transected 7–10 mm distal to the DRG so that the discharge activities of the dorsal root fibers would originate primarily from the DRG region and not from peripheral sources. During recording the L4–L5 pool was filled with warm Krebs solution (35–37°C) containing (in mM): NaCl 150, KCl 5, CaCl_2_ 2, MgCl_2_ 1, D-glucose 10 and HEPES 10, with the pH adjusted to 7.4.

The L1–L2 pool was filled with warm paraffin oil (35–37°C). Under a microscope, a microfilament (20–50 µm in diameter) and presumably including up to a few nerve fibers was isolated from the dorsal root and cut off. The proximal end was placed on a fine platinum electrode (29 µm in diameter) for electrophysiological recording of DRG single fiber activities. The firing patterns of a single fiber were displayed on a memory oscilloscope (VC-11, Japan) and recorded via an A/D board to a computer hard drive and stored for offline analysis. Unit activities with identical wave forms were selected as single fiber activities [24], [28].

Riluzole (Sigma, USA) was dissolved in dimethyl sulfoxide (DMSO) as a stock solution and kept frozen; it was diluted in ACSF before the experiments. Unit discharges were recorded in the presence and absence of riluzole or vehicle for at least another 3 min. The percentage changes in discharge rate were calculated as: (maximal discharge rate - baseline rate)/baseline rate×100%. The changes in single fiber firing rates were considered significant if the differences were 15% or greater [28].

#### 3.2 Intracellular recording of SMPOs in DRG neurons

In order to examine the effects of riluzole on these oscillations, DRG neurons from CCD animals were intracellularly recorded *in vivo* using sharp electrodes. The compressed DRG was exposed under sodium pentobabital anesthesia (40 mg/kg, i.p.). A small pool was formed surrounding the CCD ganglion and filled with warm paraffin oil, as described above. Recording microelectrodes were pulled from borosilicate glass tubes using a microelectrode puller (P97, Sutter Instruments, USA). After being filled with 3 M potassium acetate the microelectrodes had a final resistance of 40–60 MΩ. DRG neurons were impaled under visual control by advancing the microelectrode at 4–8 µm steps, with application of a small capacitance buzz when necessary.

The conduction velocity was measured by delivering a brief electrical pulse to the sciatic nerve through a stimulus isolator. DRG neurons were categorized by their conduction velocities [29]. Only A-type neurons with conduction velocities of 15.0–35.1 m/s having stable resting membrane potentials below −50 mV and/or spontaneous activity were selected for further investigation. Recordings were terminated if the resting membrane potential dropped more than 10% below control values. Riluzole (80 µM) was locally applied after control recordings.

#### 3.3 Whole-cell patch recording of sodium currents in DRG neurons

Three to eight days after the CCD surgery, the animals that showed the positive behavioral responses described above were selected for further electrophysioloical recordings. After these animals were anesthetized with sodium pentobarbital (50 mg/kg, i.p.), the compressed L5 DRGs were carefully removed from the vertebral column and placed in cold oxygenated ACSF. The ACSF contained (in mM): NaCl 125, KCl 2.5, NaH_2_PO4 1.2, MgCl_2_ 1.0, CaCl_2_ 2.0, NaHCO_3_ 25, and D-glucose 10. The connective tissue was gently removed under a microscope and the ganglia were digested with a mixture of 0.4 mg/ml trypsin (Sigma) and 1.0 mg/ml A-type collagenase (Sigma) for 40 min at 37°C while agitated by gentle bubbling with 95% O_2_ and 5% CO_2_. Finally, the ganglion was transferred into a holding chamber containing normal ACSF bubbled with 95% O_2_ and 5% CO_2_ at 26°C [23].

Recording electrodes had resistances of 4–8 MΩ after being filled with an internal solution. To measure membrane potentials and the effects of riluzole on the SMPOs of A-type DRG neurons, the internal solution contained (in mM): KCl 140, MgCl_2_ 2, HEPES 10, Mg-ATP 2, with the pH adjusted to 7.4. To examine the effects of riluzole on sodium currents in A-type DRG neurons, recording electrodes were filled with an internal solution containing (in mM): CsCl 110, NaCl 5, MgCl_2_ 3, CaCl_2_ 1, EGTA 3 and HEPES 40, adjusted with Tris buffer to pH 7.4. The bath solution consisted of (in mM): NaCl 100, TEA-Cl (tetraethylammonium chloride) 40, KCl 3, MgCl_2_ 1, CaCl_2_ 1, D-glucose 10, HEPES 10, BaCl_2_ 1, CsCl 1, 4-AP (4-aminopyridine) 2 and CdCl_2_ 0.1, with the pH adjusted to 7.4 [30].

The ganglion was kept in the holding chamber for at least 1 hr before being transferred to the recording chamber. During recording, the ganglion was kept submerged and perfused with warm (32°C) ACSF saturated with 95% O_2_ and 5% CO_2_. Individual neurons were visualized with a 40× water-immersion objective under a microscope (BX51WI; Olympus) equipped with infrared differential interference contrast optics. Whole-cell current and voltage recordings were carried out using a Multiclamp 700B amplifier (Molecular Devices, USA). The signals were digitized at 10 kHz by a 1320 A/D board (Molecular Devices, USA) and stored in a computer hard drive for offline analysis. P-clamp 9 software (Molecular Devices, USA) was used for data acquisition and analysis. Typically, a gigaohm seal was formed by a small negative pressure, and whole-cell recording was established by rupture of the cell membrane with further negative pressure or a buzz signal, or a combination of both. Membrane potential was held at −60 mV under voltage clamp. Neurons that showed resting membrane potentials below −50 mV along with overshooting action potentials were selected for further study. Recordings were terminated if the R_a_ increased or resting membrane potential dropped by 20% or more from control levels.

Sodium current amplitude was transformed into conductance using the Ohm's law in the form: G = I/(V−E_Na_), where V is the test potential and E_Na_ is the Na^+^ equilibrium potential calculated using the Nernst equation. Conductance was normalized, plotted against V and fitted with a Boltzmann function of the form: G/G_max_ = 1/{1+exp[(V−V_1/2_)×k^−1^ ]}, where V_1/2_ is the half-activation voltage and k is the slope factor.

### 4. Statistical analysis

All values were expressed as mean ± SEM. Statistical evaluations were performed using Statistical Product and Service Solutions (SPSS) software (paired t-test, non-paired t-test, repeated measure, and one-way AVOVA methods) with the significance criterion set at P = 0.05.

## Results

### 1. Mechanical allodynia in CCD animals

During the behavioral test period which lasted for up to two weeks, all animals appeared to be gaining weight, which suggests that they were in good health. Animals were usually well groomed and exhibited no self-inflicted wounds. No abnormal gait or posture was observed in the control group. However, all tested rats post-CCD surgery developed varying degrees of gait and postural abnormality. They were often seen to lift the ipsilateral hindpaw from the ground and then hold it in a protected position next to the flank while standing or sitting. When the affected hindpaw was touching the ground, the animals often reduced the weight placed on it by leaning to the other side or by sitting on the opposite haunch. These behaviors appeared as early as one day after the CCD surgery, and all animals showed such behaviors within the first two weeks, as reported earlier [24], [25]. No control or test animals exhibited any signs of autotomy or abnormal nail growth. However, the CCD rats used for subsequent electrophysiological recordings showed clear behavioral indications of allodynia, as reported previously [24], [25].

**Figure 1:**
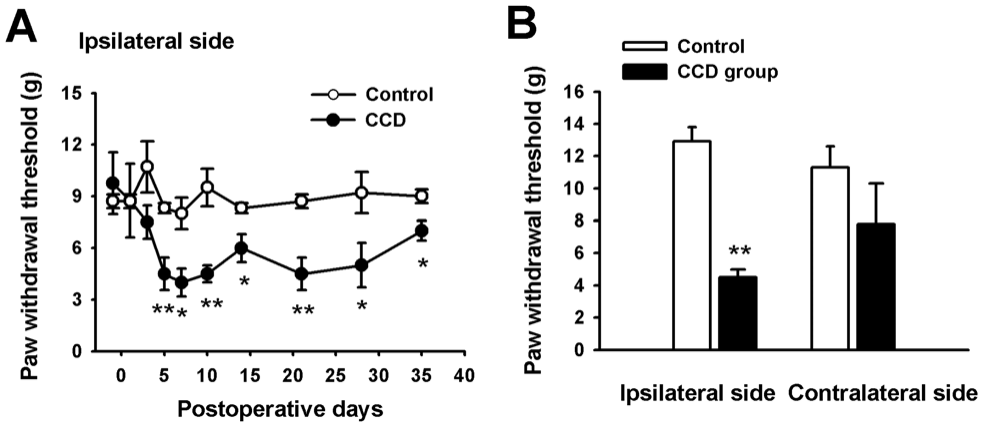
Mechanical allodynia in CCD rats. (A) Time course of changes in the withdrawl threshold of the ipsilateral hindpaw for control (n = 6, open circles) and CCD (n = 4, closed circles) rats. The mechanical threshold for withdrawl of the ipsilateral hindpaw was significantly lower in the chronically compressed rats (* P<0.05 or **P<0.01 compared with the average for the ipsilateral hindpaw of the control group). (B) Average ipsilateral and contralateral paw withdrawl thresholds for the control and CCD groups on the postoperative 5 days.

As shown in Figure 1A, the thresholds for paw withdrawal in control animals were relatively stable (8.5±0.4 g, n = 6), while the values for the same tests on the contralateral side were slightly decreased (Fig. 1B), but not to a statistically significant extent (non-paired t-test, P>0.05). However, the thresholds for paw withdrawal on the ipsilateral side of the CCD animals were clearly reduced starting on the day after the CCD surgery (one-way AVOVA method, P<0.05). The reductions became statistically significant from day 5 on and remained low until the end of the tests (4.0±0.8 g, n = 4; non-paired t-test, P<0.05, Fig. 1A). These behavioral responses of the CCD animals are characteristic of mechanical allodynia. Similar results have been reported by our group [24] and by others [25].

### 2. Inhibitory effects of *I_NaP_* blocker riluzole on the spontaneous activity of A-type fibers from compressed DRG neurons

A total of 53 spontaneously discharging A-type single fibers of the L5 DRG were recorded from 38 CCD ganglions in intact animals. CCD fibers/neurons can be classified into two classes based on the dynamic features of their spontaneous activities [28]. The periodic class is characterized by the interspike intervals that appear repeatedly at regular intervals, and 21 of 53 fibers (39.6%) fell into this category. Fibers in the non-periodic class have an irregular pattern of interspike intervals, and 32 of 53 fibers (60.4%) were included in this group.

**Figure 2:**
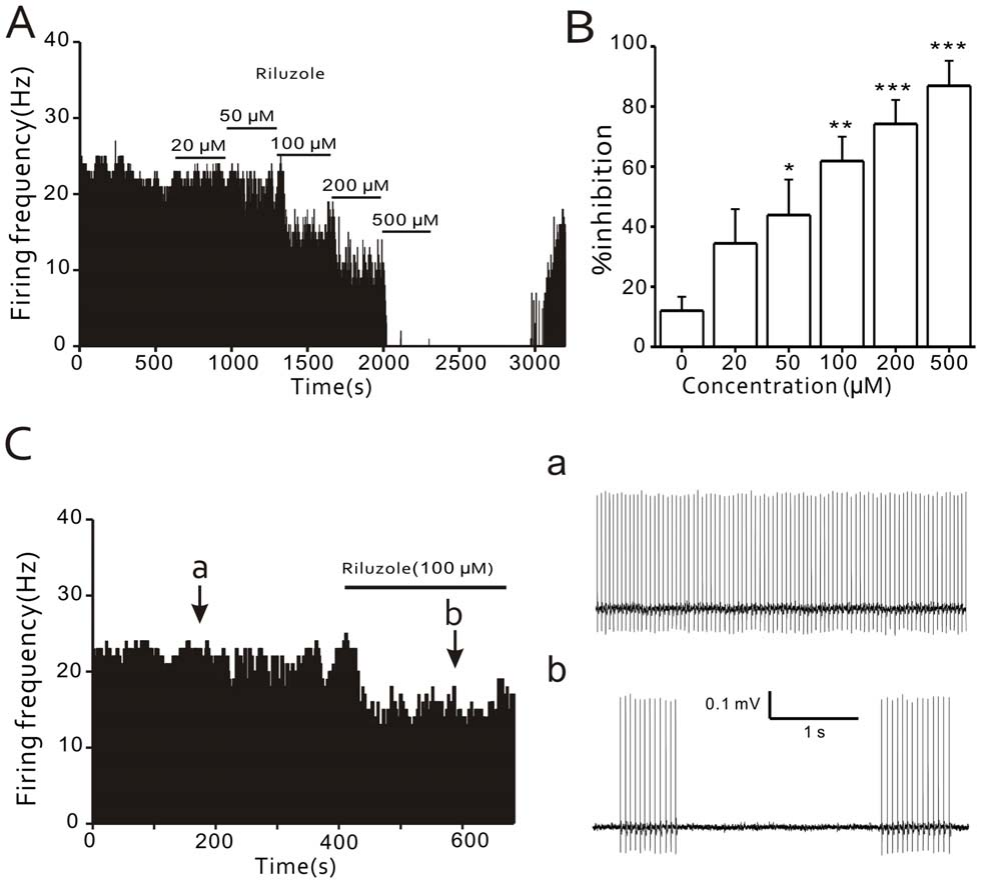
Effects of riluzole on the spontaneous activity of A-type neurons in the compressed DRG. (A) Time histogram showing that local application of the *I_NaP_* blocker riluzole reduces the basal firing rate of an A-type fiber in a dose-dependent manner (n = 6; P<0.05). (B) Percent inhibition of spontaneous activity in A-type fibers with respect to the concentration of locally-applied riluzole (µM; n = 6; *P<0.05, **P<0.01, ***P<0.001). (C) Time histogram showing the suppression of spontaneous activity induced by riluzole (100 µM) in an A-type afferent fiber from a compressed DRG. Expanded traces in right panel show firing patterns before (a) and during (b) the drug application.

Basal firing rates normally fluctuated from 0.6% to 14.2% of the average control value (6.4±0.5%, n = 53). Five minutes after local application of the *I_NaP_* blocker riluzole (100 µM) to the DRG region, rates of spontaneous activity were reduced in all cases (n = 6). Figure 2A shows one such recording, in which firings were reversibly abolished after the riluzole application. The results of similar recordings can be summarized as showing that the spontaneous activity of A-type CCD fibers is suppressed by riluzole in a dose-dependent manner (n = 6, Fig. 2B). Again, the riluzole-mediated inhibition of the spontaneous activity in A-type CCD fibers was shown to be reversible in most cases, as the spontaneous activity resumed in 78.6% (22/28) of the fibers within 20 min after washout (Fig. 2A). In the presence of 0.1% DMSO, the discharge rate of A-type fibers from compressed DRG neurons was 19.9±2.9 Hz (n = 4), which is not significantly different from the discharge rate of A-type fibers in the absence of the vehicle (paired t-test, P>0.05).

### 3. Inhibitory effects of riluzole on SMPOs in compressed A-type DRG neurons

#### 3.1 Inhibitory effects of riluzole on spontaneous SMPOs at the resting membrane potential

**Figure 3:**
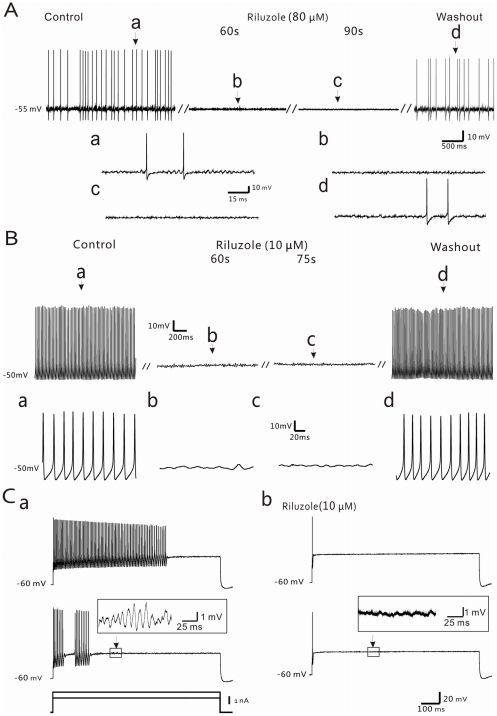
Effects of riluzole on the spontaneous activity and SMPOs of A-type neurons in the compressed DRG. (A) Intracellullar sharp electrode recordings from an A-type DRG neuron *in vivo*, showing spontaneous activity and SMPOs under control conditions (a), after local application of riluzole (80 µM; b,c), and during washout (d). The spikes have been truncated. Inserted segments (a–d, lower panel) are expansions of the original traces at the times indicated and show details of the spikes and SMPOs. (B) Whole-cell patch recordings from an A-type DRG neuron *in vitro*. Spontaneous activity (a) was significantly inhibited in the presence of riluzole (10 µM; b,c) and restored after washout (d). The spikes have been truncated. Inserted segments (a–d, lower panel) are expansions of the original traces at the times indicated. (C) Whole-cell patch recordings from an A-type DRG neuron *in vitro*. (a) The cell responded to depolarizing current pulses of 1.7 nA (upper panel) and 2.4 nA (lower panel) with repetitive firing and SMPOs (insertion). (b) In the presence of 10 µM riluzole (upper panel), both the repetitive firing and SMPOs (insertion) induced by depolarization were abolished; however, a single action potential was still evoked immediately after application of the current pulse.

The SMPOs observed in DRG neurons of CCD animals can be considered a useful electrophysiological indication of neuronal injury. To examine whether riluzole affects SMPOs and would thereby block such downstream effects, DRG neurons in CCD animals were recorded *in vivo* using sharp electrodes. Upon penetration, most of these DRG neurons were silent and had a relatively stable membrane potential (−61.3±0.9 mV, n = 30), but some (7/63, 11.1%) displayed sinusoidal SMPOs (78.2±3.4 Hz, n = 12) and spontaneous activity at the resting membrane potential. After a 5 min period of baseline recording from 4 of such neurons, riluzole (80 µM) was locally applied onto the DRG. It was found that the resting membrane potential remained stable in all of these cells, but that the frequency of the spontaneous activity gradually decreased and finally disappeared in about 60 s. The SMPOs showed similar changes, decreasing in amplitude and then disappearing. The inhibition of riluzole on spontaneous activity and SMPOs was largely recovered following washout in all the tested neurons (Fig. 3A).

#### 3.2 Inhibitory effects of riluzole on the spontaneous activity *in vitro*

Few if any DRG neurons recorded using whole-cell patch-clamp methods displayed spontaneous activity following the compression of the DRG. The effects of riluzole were examined in these neurons by the bath application of the drug (10 µM) to three DRG neurons *in vitro*, after which the spontaneous activity slowed down and eventually was eliminated (Fig. 3B).

#### 3.3 Inhibitory effects of riluzole on SMPOs at different levels of depolarization

Using whole-cell patch-clamp methods, about a third of the recorded DRG cells (10/28, 35.7%) displayed high frequency sinusoidal SMPOs and repetitive discharges during the injection of 800 ms depolarizing current pulses. The effects of riluzole were examined in five of these neurons by the bath application of the drug (10 µM). It was found that the SMPOs were eliminated and that the repetitive firing slowed down and eventually stopped 3 min after the drug application. It is interesting to note that although riluzole largely abolished the evoked SMPOs and the repetitive firing, these DRG neurons still responded to the same depolarizing current pulses with a single action potential at the initial phase of the current pulse, indicating that these cells are still capable of generating action potentials in the presence of riluzole (Fig. 3C). In all the neurons tested, the SMPOs and repetitive firing were largely recovered after riluzole washout.

### 4. Increased *I_NaP_* in injured A-type DRG neurons

Using whole-cell patch-clamp methods, 114 large- and medium-sized neurons (≥35 µm in diameter) were recorded in *in vitro* DRG preparations taken from CCD animals. These neurons had a stable resting potential of −55.7±0.9 mV (n = 80), a membrane resistance of 89.6±5.3 MΩ (n = 80), and a membrane capacitance of 92.4±3.3 pF (n = 80).

**Figure 4:**
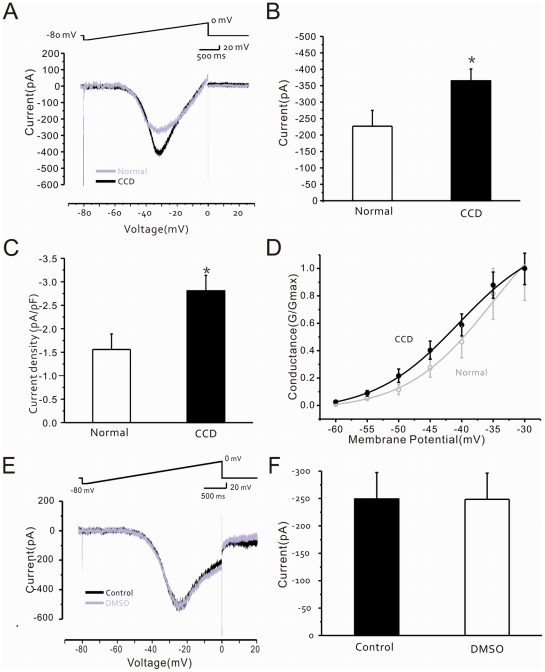
Enhanced *I_NaP_* in A-type neurons of the compressed DRG. (A) Traces of *I_NaP_* in A-type neurons from control (normal; grey line) and compressed (CCD, solid line) DRGs (bottom) as induced by increasing applied voltage (top). (B) Bar graph showing that the average peak *I_NaP_* is significantly greater in neurons recorded from compressed DRGs (CCD; n = 31) compared to the average peak value from control group recordings (normal; n = 26; *P<0.05). (C) Bar graph showing that average peak *I_NaP_* current density is significantly greater in compressed DRG neurons (n = 31) compared to the average peak value from control group recordings (n = 26; *P<0.05). (D) Steady state activation curves for *I_NaP_* conductance with respect to voltage in control and compressed DRG neurons as fit with a Boltzmann distribution equation. The differences are not statistically significant. (E) Traces of *I_NaP_* in a compressed DRG neuron in the absence (control, solid line) and presence (DMSO, grey line) of 0.1% DMSO (bottom) as induced by increasing applied voltage (top). (F) Bar graph showing that average peak *I_NaP_* in compressed DRG neurons (n = 7) in the presence of 0.1% DMSO is not significantly different from the average peak *I_NaP_* in the absence of DMSO (control, filled; n = 7; P>0.05).

In these experiments, the glass recording pipettes were filled with a Cs^+^-based internal solution, while K^+^ and Ca^2+^ channel blockers were added to the bath. Under a holding potential of −60 mV, *I_NaP_* was recorded in normal DRG neurons by applying a 3 s depolarization ramp current from −80 to 0 mV [30]. The inward sodium current was induced at potentials of −60 to −50 mV, reached a peak at −35 mV, returned to control levels at −20 mV (Fig. 4A), and was sensitive to a low dose of TTX (100 nM, not shown). In injured A-type DRG neurons, the average current density of I_NaP_ was also significantly increased (CCD group: 2.8±0.3 pA/pF, control group: 1.6±0.3 pA/pF, non-paired t-test, P<0.05; Fig. 4A–4C). The activation curves of *I_NaP_* in control and compressed DRG neurons were both fit with a Boltzmann distribution equation; the differences were not statistically significant (Fig. 4D).

### 5. Selective inhibitory effect of riluzole on *I_NaP_* in injured A-type DRG neurons

**Figure 5:**
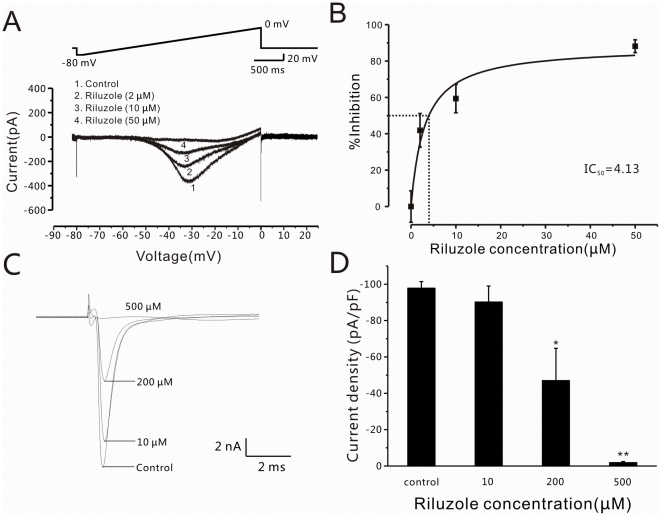
Effects of riluzole on *I_NaP_* and *I_NaT_* in A-type neurons of the compressed DRG. (A) Traces of *I_NaP_* (bottom) induced by increasing applied voltage (top) in a compressed DRG neuron in the absence (control) and presence of riluzole (2, 10, 50 µM). (B) Dose-inhibition curve showing effect of locally applied riluzole on *I_NaP_* in compressed DRG neurons (n = 5; IC_50_ = 4.3 µM). (C) *I_NaT_* traces in a DRG neuron evoked by applied depolarization (−20 mV) under control conditions and in the presence of riluzole (10, 200, 500 µM). (D) Bar graph showing that average peak *I_NaT_* current density (pA/pF) in the presence of riluzole (10, 200, 500 µM) is significantly decreased in neurons recorded from compressed DRGs (*P<0.05, **P<0.01).

The average current peak of *I_NaP_* in injured A-type DRG neurons was 249.1±48.3 pA or 248.4±47.9 pA (n = 4) in the absence or presence of DMSO (0.1%), respectively, a difference which is not statistically significant (paired t-test, P>0.05; Fig. 4E&4F). Riluzole significantly inhibited the *I_NaP_* of injured A-type DRG neurons in a dose-dependent manner. At doses as low as 2 µM, the drug clearly reduced the peak value of *I_NaP_* by ∼40% (n = 5, non-paired t-test, P<0.05; Fig. 5A), and the IC_50_ for riluzole inhibition of the *I_NaP_* was 4.1 µM (n = 5; Fig. 5B).

To determine the specificity of this riluzole-induced inhibition in injured A-type DRG neurons, we next examined the effects of the drug on the TTX-sensitive transient sodium current (*I_NaT_*). In whole-cell recordings performed under voltage-clamp, *I_NaT_* was evoked by depolarization voltage steps (Fig. 5C). Riluzole was then bath-applied at 10, 200 and 500 µM, and the percent inhibition of the *I_NaT_* peak amplitude in each neuron was plotted against the different voltage steps (Fig. 5D). The inhibition of *I_NaT_* at 200 and 500 µM riluzole was 58.9±6.5% (n = 4, paired t-test, P<0.05) and 82.0±1.7% (n = 3, paired t-test, P<0.001), respectively.

## Discussion

The primary finding of the present study is that a local application of the *I_NaP_* blocker riluzole to the DRG selectively reduces the *I_NaP_* and *I_NaP_*-associated SMPOs of injured DRG neurons in the CCD animal model of neuropathic pain. The *I_NaP_* is enhanced in medium- and large-sized DRG neurons of the compressed DRG.. Our data shows for the first time that the *I_NaP_* and *I_NaP_*-associated SMPOs of injured DRG neurons are important to the spontaneous activity of primary afferents in neuropathic pain states. Hence, the blockade of *I_NaP_* in DRG neurons may play an anti-nociceptive role at the primary afferent level.

### 1. Spontaneous activity in neuropathic pain states

In our animal model, a chronic and steady compression is ipsilaterally applied to the DRG in rats (CCD model), causing the animals to show behaviors typical of mechanical allodynia ipisilaterally [24]. Ectopic spontaneous activity plays an important role in maintaining central sensitization and neuropathic pain. Moreover, the level of ectopic discharge is generally well correlated with the degree of pain behavior in neuropathic animals [31]. Our *in vivo* experiment demonstrate that the spontaneous activity of large diameter primary afferent fibers is inhibited by the *I_NaP_* blocker riluzole in a dose-dependent manner and is completely abolished at a concentration of 500 µM. This concentration of riluzole was slightly higher than that of the riluzole used in our *in vitro* whole-cell patch-clamp experienments of the present study, due to the rapid circulation of blood within the DRG *in vivo*. These results indicate that the *I_NaP_* blocker riluzole inhibits the spontaneous activity of compressed type-A DRG neurons, and hence reduces pathological pain signals originating in the periphery.

### 2. SMPOs in A-type DRG neurons

Previous work has shown that low levels of TTX also suppress SMPOs and abolish ectopic spontaneous activity in injured DRG neurons [32], [33], [34], indicating that SMPOs of injured DRG neurons are essential to the generation of abnormal spontaneous activity and pain behaviors. The fact that peripheral receptors and large parts of the axons were removed in our preparations suggests that SMPOs must be generated in the soma or in proximal segments of DRG cell axons. Hence, pathological hyperexcitability may originate not only at receptors in the skin but also in the somatic region of primary afferent neurons. Our recent work has shown that analgesic drugs such as gabapentin and lidocaine have inhibitory effects similar to those of riluzole on *I_NaP_* [22], [23]. The results reported here further demonstrate that *I_NaP_* plays an important role in the generation of SMPOs that occur in injured A-type DRG neurons after compression of the DRG. In the rat trigeminal mesencephalic nuclei, *I_NaP_* is responsible for the enhancement of SMPOs and also participates in the generation of bursting in central neurons [19]. Taken together, these studies strongly suggest that injury and inflammation may cause large- and medium-sized DRG neurons to increase a TTX-sensitive *I_NaP_* and its associated SMPOs. The latter may in turn facilitate the generation of ectopic spontaneous activity, an important basis for pathological pain signals in the periphery under neuropathic pain states. Using sharp electrode recordings *in vivo* and whole-cell patch-clamp recordings *in vitro*, we observed sine wave-like SMPOs in DRG neurons. Moreover, although repetitive firing and SMPOs were abolished by riluzole, DRG neurons still responded to intracellular current injection with a single full-sized spike, demonstrating that they are still capable of firing action potentials under riluzole.

### 3. *I_NaP_* in A-type DRG neurons

The vast majority of pain studies at the peripheral level have focused on small DRG neurons (<25 µm in diameter) and their fibers. However, in cases of allodynia the non-nociceptive stimuli associated with these cells become nociceptive, and almost any type of stimulus may cause pain. It is a reasonable hypothesis that medium- and large-sized primary afferent fibers and their DRG neurons are responsible for allodynia [35], [36]. The present study has focused on these neurons and their fibers in a CCG model to further characterize allodynia's cellular mechanisms.

*I_NaP_* is a TTX-sensitive current that is activated in the subthreshold voltage range and is slowly inactivating. Such dynamics enable neurons to amplify their responses to synaptic inputs, thereby driving them to spiking or repetitive firing [9], [11]. To measure *I_NaP_* directly, we injected ramp current into the recorded cells while isolating the *I_NaP_* by adding the non-selective K^+^ channel blocker cesium to the internal solution and the non-selective Ca^2+^ channel blocker cadmium to the bath [30]. Our results show that *I_NaP_* is indeed present in normal A-type DRG neurons and is blocked by the presence of a low concentration of TTX (100 nM). Moreover, after induction of the ganglion compression characteristic of the CCD model, *I_NaP_* was significantly increased in A-type DRG neurons. *I_NaP_* activation curves for compressed DRG neurons were not significantly different from those of the control group. These results suggest that increased *I_NaP_* contributes to the hyperexcitability of injured A-type DRG neurons.

There is evidence that under experimental pain conditions, at least two types of Na^+^ channels, Na_v_1.7 and Na_v_1.8, are upregulated and downregulated concurrently with corresponding effects on TTX-sensitive and TTX-insensitive currents [37]. The sodium channel Na_v_1.7 is expressed predominantly in DRG and sympathetic ganglion neurons [38], [39], [40], [41], specifically in most functionally-identified DRG nociceptive or small neurons [42]. This channel has been proposed as a molecular gatekeeper of pain detection at peripheral nociceptors [43]. Na_v_1.7 is slowly inactivated with dynamics similar to those of *I_NaP_* [44]. The expression of Na_v_1.7 in large- and medium-sized DRG cells, in addition to its upregulation concurrent with changes in *I_NaP_*, have been observed in a model of diabetic neuropathy [45]. There is also evidence that increased *I_NaP_* determines cortical hyperexcitability in a genetic model of amyotrophic lateral sclerosis [11].

### 4. Potential anti-nociceptive effects of *I_NaP_* blocker riluzole

Animal studies have shown that riluzole reduces the development of mechanical and cold hyperactivities in a neuropathic pain model through its pronounced suppression of glutamate and aspartate levels in the spinal dorsal horn [46]. Riluzole also attenuates formalin-induced flinching behaviors that follow from its blocking effects on sodium channels, although a specific site of this action has not been demonstrated [47]. In the rat, local injection of riluzole into the ventral posterolateral thalamic nuclei was found to reduce carrageenan-induced mechanical hyperalgesia due to a decrease in glutamate release [48]. In addition, riluzole was reported to induce anti-nociceptive effects along with a general anesthetic state, probably by blocking glutamatergic neurotransmission [49]. However, clinical research has shown that oral administration of riluzole does not affect thermal and mechanical hyperalgesia in patients with inflammatory pain [50] or alleviate allodynia and mechanical hyperalgesia in neuropathic pain patients [51].

These controversial effects of riluzole in clinical application and animal research call for simplified model systems to explore its basic mechanisms at cellular and molecular levels. To avoid the complexity of riluzole's effects on the CNS, we have systematically investigated its effects on DRG neurons as representing the first stage of the peripheral pain sensory system. We have shown that riluzole selectively inhibits the *I_NaP_* of injured A-type DRG neurons, thereby suppressing their SMPOs and ectopic spotaneous activity. Those results suggest that the *I_NaP_* of DRG neurons can be a potential target for analgesia at the peripheral level.

Our results indicating that riluzole may excert an anti-allodynia effect in the DRG after nerve injury are also consistent with reports that riluzole decreases the development of mechanical allodynia in a rat model of neuropathic pain [46] and that the drug attenuates nociceptive responses in a formalin-induced inflammatory model [47]. Clinically, however, riluzole does not affect thermal hyperalgesia in the inflammatory pain induced by heat stimuli [50]. These results strongly suggest that riluzole selectively reduces mechanical allodynia under neuropathic pain states, but is not as effective in treating other types of pain that are conveyed via thin afferent fibers. Compared with the anti-convulsant agents gabapentin and lamotrigine, lower doses of riluzole were more effective in relieving mechanical hypersensitivity. The effects of the drug were also long-lasting, extending up to 12 days after systematic administration [46]. Apparently, riluzole is much more efficient than these other agents in reducing mechanical hypersensitivity under conditions of neuropathic pain. The discrepancy of its clinical ineffectiveness may be due to the use of insufficient doses of the drug at local sites. Our recent work showing that *I_NaP_* is suppressed by gabapentin and low doses of lidocaine in compressed DRG neurons [22], [23], as well as the inhibitory effects of riluzole in the same CCG model shown here, indicate that the I_NaP_ of injured DRG neurons remains a potential target for analgesia at the peripheral level.

From a clinical point of view, most pain symptoms in peripheral neuropathy are of peripheral origin [52]. The gateway function of the DRG suggests that the *I_NaP_* of DRG neurons could be a potential target in treating patients with peripheral pain symptoms. Our results demonstrate that local application of *I_NaP_* blocker riluzole within the DRG could be specific and effective as a peripherally-acting analgesic agent that does not penetrate the blood-brain barrier and is therefore free of central nervous system side effects [53].
